# Functional characterization of the CRY2 circadian clock component variant p.Ser420Phe revealed a new degradation pathway for CRY2

**DOI:** 10.1016/j.jbc.2023.105451

**Published:** 2023-11-10

**Authors:** Gizem Cagla Parlak, Ibrahim Baris, Seref Gul, Ibrahim Halil Kavakli

**Affiliations:** 1 Department of Molecular Biology and Genetics, Koc University, Istanbul, Turkiye; 2 Institute of Life Sciences and Biotechnology, Bezmialem Vakif University, Beykoz, Turkiye; 3 Department of Chemical and Biological Engineering, Koc University, Istanbul, Turkiye

**Keywords:** cryptochrome 2, SNP, lysosomal degradation pathway, circadian rhythm, proteasomal degradation pathway

## Abstract

Cryptochromes (CRYs) are essential components of the circadian clock, playing a pivotal role as transcriptional repressors. Despite their significance, the precise mechanisms underlying CRYs’ involvement in the circadian clock remain incompletely understood. In this study, we identified a rare CRY2 variant, p.Ser420Phe, from the 1000 Genomes Project and Ensembl database that is located in the functionally important coiled-coil-like helix (CC−helix) region. Functional characterization of this variant at the cellular level revealed that p.Ser420Phe CRY2 had reduced repression activity on CLOCK:BMAL1−driven transcription due to its reduced affinity to the core clock protein PER2 and defective translocation into the nucleus. Intriguingly, the CRY2 variant exhibited an unexpected resistance to degradation *via* the canonical proteasomal pathway, primarily due to the loss of interactions with E3 ligases (FBXL3 and FBXL21), which suggests Ser-420 of CRY2 is required for the interaction with E3 ligases. Further studies revealed that wild-type and CRY2 variants are degraded by the lysosomal-mediated degradation pathway, a mechanism not previously associated with CRY2. Surprisingly, our complementation study with *Cry1*^*−/−*^*Cry2*^*−/−*^ double knockout mouse embryonic fibroblast cells indicated that the CRY2 variant caused a 7 h shorter circadian period length in contrast to the observed prolonged period length in *CRY2*^*−/−*^ cell lines. In summary, this study reveals a hitherto unknown degradation pathway for CRY2, shedding new light on the regulation of circadian rhythm period length.

Circadian rhythms, endogenous timekeeping mechanisms found in almost all organisms, play a vital role in maintaining homeostasis and promoting survival in response to environmental changes (1). These rhythms are governed by the circadian clock, an internal oscillator that orchestrates various behaviors, biochemical processes, and physiological activities in alignment with daily cycles (1). Disruptions of circadian rhythm are associated with various types of diseases, such as metabolic and sleep disorders (2, 3, 4).

The circadian clock is generated by cell-autonomous transcriptional/translational feedback loops (TTFLs) and is evolutionarily conserved across species (5, 6). In mammals, the primary TTFL consists of positive and negative arms, where BMAL1 and CLOCK form a heterodimer in the positive arm and bind to E-box regions on clock-controlled genes (CCGs) including *Period*s (*Per*s) and *Cryptochrome*s (*Cry*s). As the levels of CRYs and PERs rise in the cytoplasm, they translocate to the nucleus, where they interact with the CLOCK:BMAL1 dimer, suppressing the transcription of CCGs through mechanisms involving blocking or displacement, depending on the presence of PER (7, 8). These interactions constitute the negative arm of the TTFL. Furthermore, auxiliary feedback loops are essential for the precise generation and regulation of circadian rhythms in mammals (6).

CRYs, characterized by their PHR domain and extended C-terminal domain, are crucial for the maintenance and amplitude of circadian rhythmicity through interactions with core clock proteins (9, 10, 11). While the N-terminal region is critical for rhythmicity, a variable C-terminal tail contributes to circadian rhythm amplitude (10, 11). Within the PHR domain, there are three subdomains: a primary pocket (homologous to the FAD-binding domain in photolyase), a secondary pocket (homologous to the chromophore-binding domain in photolyase), and a coiled-coil-like helix (CC−helix). The secondary pocket plays a crucial role in localizing CRY2 to the nucleus and sustaining circadian rhythm (12). The CC*−*helix of CRYs interacts with the C-terminal of PER2 (13) and is essential for the binding to E3 ubiquitin ligases, FBXL3 and FBXL21, which mediate CRY degradation. These ligases compete with PER2 for binding around the region involving the CC−helix and the C-terminal tail of CRY, thus influencing CRY stability (14, 15, 16). Deletion of the CC−helix and nuclear localization signals (NLS) in CRY1 tails leads to exclusive cytoplasmic localization, highlighting its role in proper nuclear translocation (17). Furthermore, this region is critical for the interaction between the CRY1 PHR domain and its extended C-terminal domain with CLOCK:BMAL1 (18, 19). Besides the activity of E3 ligases and proteasomal degradation, clock proteins are subject to lysosomal-mediated degradation, known as macroautophagy (20). CRY1 contains a light chain 3 (LC3)-interacting region (LIR) motif, enabling interaction with LC3 and directing the protein toward autophagic degradation (21). Autophagic degradation of CRY1 activates hepatic gluconeogenesis, highlighting the significance of autophagy in regulating CRY1. Interestingly, this same study reported no change in the levels of liver CRY2 upon autophagy inhibition (20). Therefore, understanding the structure and function of CRYs and their interactions with other proteins is crucial for deciphering the mechanisms underlying circadian rhythmicity and its impact on various physiological processes.

In this study, we performed molecular and functional characterizations of the CRY2 p.Ser420Phe variant, located within the CC-helix of the PHR domain. This region is crucial for interactions with other core clock proteins and E3 ligases. Our findings showed that, although the p.Ser420Phe CRY2 is unable to interact with both FBXL3 and FBXL21, it displays significantly reduced stability compared to wild-type CRY2. Consequently, we explored the lysosomal degradation pathway as an alternative mechanism by conducting a CHX chase experiment with lysosome inhibitors Pepstatin A and E64-d. This intervention increased the stability of the p.Ser420Phe CRY2 variant, providing compelling evidence that the CC-helix of CRY2 is pivotal not only for its interaction with E3 ligases but also for its degradation through the lysosomal pathway. Additionally, complementation studies in *Cry1*^*−/−*^*Cry2*^*−/−*^ double knockout mouse embryonic fibroblast cells revealed that the p.Ser420Phe CRY2 variant significantly shortened the circadian rhythm period length compared to wild-type CRY2. We have demonstrated that the p.Ser420Phe variation confers a gain of function and leads to the degradation of CRY2 *via* the lysosomal degradation pathway, thereby influencing the molecular clock.

## Results

### Determination of the repressor capacity of p.Ser420Phe CRY2 variant on CLOCK:BMAL1 transactivation

**Figure 1:**
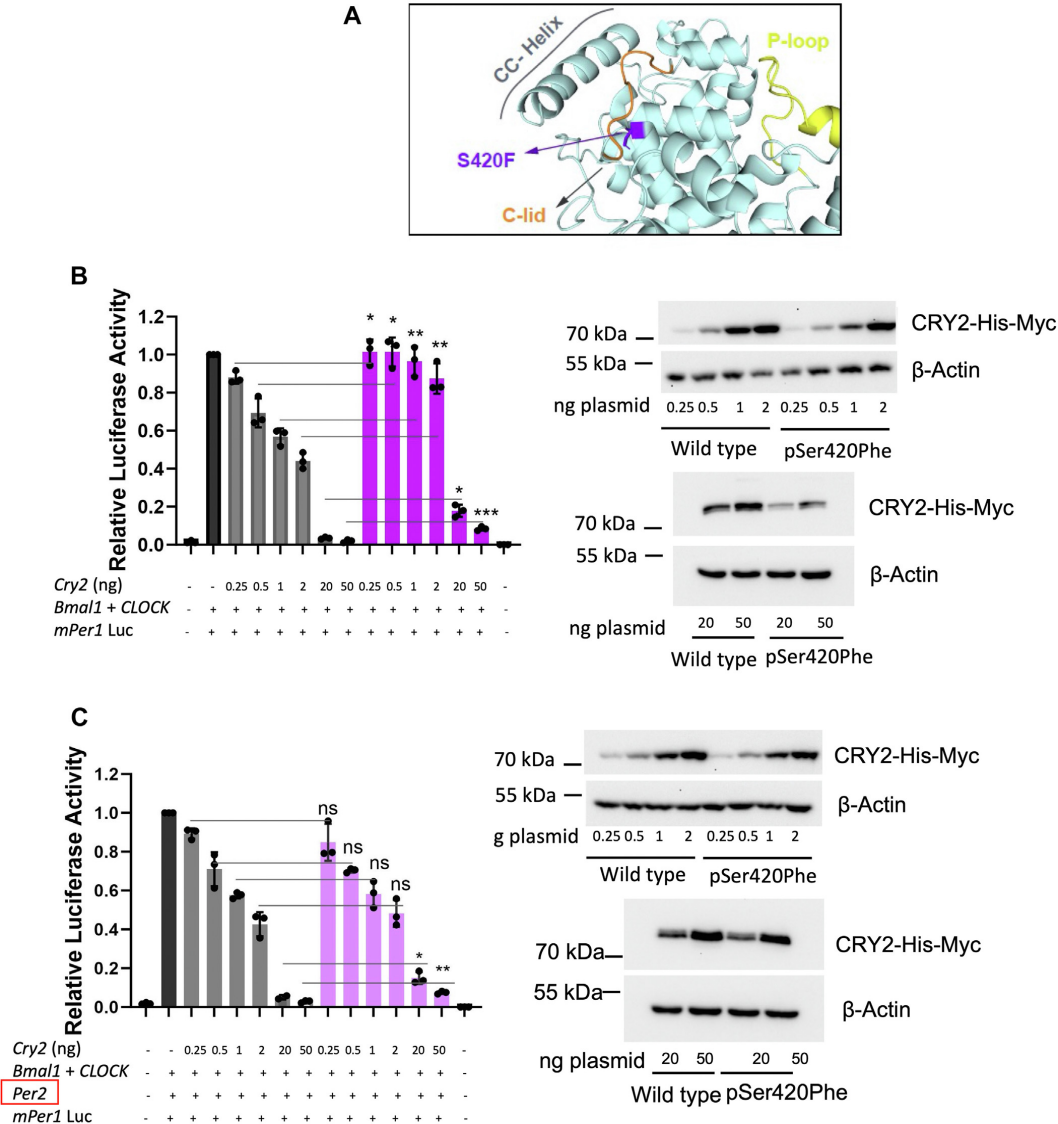
**Repression activity ofCRY2 variant CRY2.***A*, Ser420 residue (*purple*) was visualized on CRY2 as a *ribbon diagram*. The C-lid was colored *orange*, and the P-loop *yellow*. *B*, dose-dependent repression on CLOCK:BMAL1 transactivation of WT and p.Ser420Phe CRY2 without PER2 overexpression. *C*, repression activity of the WT CRY2 and CRY2 variant in the presence of PER2 overexpression. HEK293T cells were transfected with 0.25 ng, 0.5 ng, 1 ng, 2 ng, 20 ng, and 50 ng of plasmids of WT or p.Ser420Phe CRY2. Data represent the mean  ±  S.D. of three biological replicates with at least three technical replicates. The equal dose of WT and mutant CRY2 was compared, and unpaired *t* test was used for significance analysis (n = 3; ∗*p* < 0.05; ∗∗*p* < 0.01; ∗∗∗*p* < 0.001). The expression of each dose was controlled by Western blot. CRY2, cryptochrome 2; HEK293T, human embryonic kidney 293T cell line; PER2, period 2; WT, wild type.

The rare human variant c.1259C > T, known as p.Ser420Phe in CRY2 (equivalent to p.Ser419Phe in the mouse), has been identified from the 1000 Genomes Project and Ensembl database (12). Remarkably, this particular variant has not yet been linked to any discernible phenotype, leading to its classification as a "variant of uncertain significance" based on the American College of Medical Genetics and Genomics guidelines. Notably, our examination of the 3D structure of CRY2 revealed that the Serine residue at position 420 is located within the CC*−*helix, a region well-known for its functional importance in interactions with FBXL3 and FBXL21 (12, 13, 15, 16) (Fig. 1*A*).

To comprehensively explore the implications of this genetic variation on CRY2's functional attributes, we replaced Serine with Phenylalanine *via* the site-directed mutagenesis method. After confirming the mutation by Sanger sequencing, we assessed its effect on the repressive activity of CLOCK:BMAL1-driven transcription using the *Per1*-d*Luc* assay. In this assay, the *Per1* promoter is fused with a destabilized luciferase gene, and the experiment was performed in a dose-dependent manner using the HEK 293T cell line. As anticipated, increasing the dosage of the plasmid containing wild-type *Cry2* cDNA led to a dose-dependent decrease in luciferase activity, signifying the effective repression of CLOCK:BMAL1 transactivation by wild-type CRY2 (Fig. 1*B*). However, the introduction of the mutant CRY2, bearing the p.Ser420Phe alteration, resulted in significantly reduced repression of CLOCK:BMAL1 transactivation when compared to the wild type, across a wide range of doses (Fig. 1*B*). To investigate whether this difference in function between wild-type CRY2 and the variant is a result of expression or stability problems, we performed Western blot analyses on these samples. The variant exhibited reduced protein expression, particularly pronounced at higher doses (20 and 50 ng), implying that the CRY2 variant displayed lower stability compared to wild-type CRY2, potentially affecting its repressor capacity (Fig. 1*B*). Given that PER2 is known to stabilize CRYs (22), we hypothesized that introducing PER2 in the transactivation assay might enhance the stability of the variant and consequently improve its repressor activity. Therefore, we performed the *Per1-*d*Luc* assay in the presence of PER2, using a plasmid containing *Per2* cDNA. Although PER2 was able to rescue the stability of the CRY2 variant as assessed by Western blot, the CRY2 variant had reduced repressive activity at the dose of 20 and 50 ng of plasmids (Fig. 1*C*).

In summary, the assessment of the p.Ser420Phe CRY2 variant's repressor capacity on CLOCK:BMAL1 transactivation revealed significant functional differences when compared to the wild-type CRY2. While the variant displayed reduced stability and could be partially stabilized by PER2, it consistently exhibited impaired repressor activity, suggesting altered interactions with essential core clock proteins.

### Investigation of the interaction between p.Ser420Phe CRY2 with CLOCK, BMAL1, and PER2

Given that the p.Ser420Phe mutation resides within the PHR domain, responsible for binding to core clock proteins (CLOCK, BMAL1, and PER2), we started to explore how this variation affects interactions with these core clock proteins using Co-immunoprecipitation (Co-IP) assay. HEK 293 T cells were transfected with plasmid containing either wild type or variant pcDNA-*Cry2*-His-Myc plasmids along with Flag-CMV-*CLOCK*, and Flag-CMV-*Bmal1*, with or without pFLAG-CMV-*Per2* overexpression. MYC resin was used for Co-IP to precipitate CRY2-His-Myc, after which Western blot analysis was employed to quantify the presence of its binding partners: FLAG-CLOCK, FLAG-BMAL1, and FLAG-PER2.

**Figure 2:**
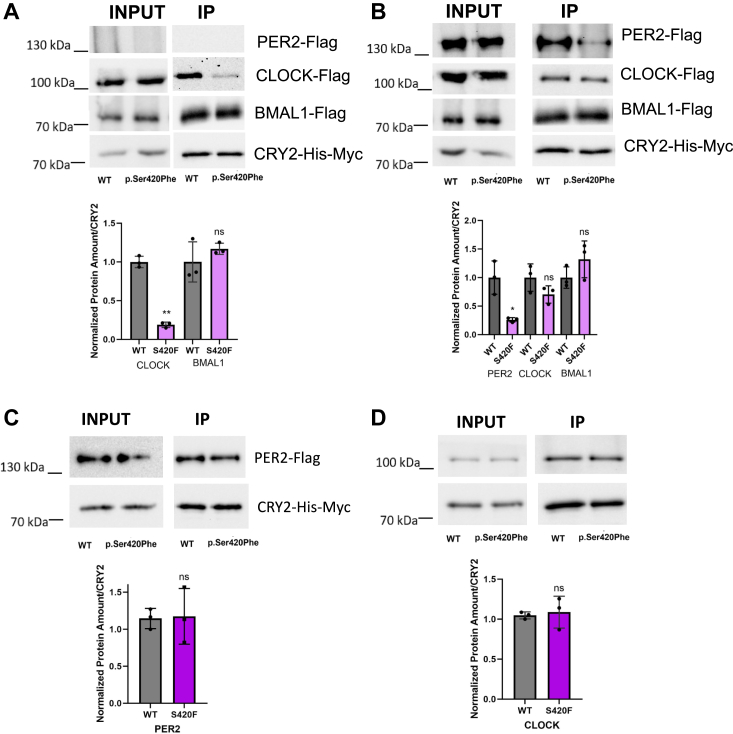
**Determination of the interaction between p.Ser420Phe CRY2 with CLOCK, BMAL1, and PER2.***A*, physical interaction of WT and p.Ser420Phe CRY2 without PER2 overexpression, and (*B*) in the presence of PER2 overexpression (n = 3). HEK293T cells were transfected with 500 ng WT or variant pcDNA-*Cry2*-His-Myc plasmids, 150 ng Flag-CMV-CLOCK, 1000 ng Flag-CMV-*Bmal1*, and 350 ng pFLAG-CMV-*Per2* (for *B*). The next day, the cells were harvested, and Co-IP was performed using MYC resin. The amount of PER2, CLOCK, and BMAL1 was determined by Western blot and divided by the amount of CRY2, normalized based on WT CRY2. *C*, physical interaction of WT and p.Ser420Phe CRY2 with Flag-CMV-PER2. *D*, physical interaction of WT and p.Ser420Phe CRY2 with pFLAG-CMV-CLOCK. Data represent the mean  ±  S.D. of three biological replicates. The unpaired *t* test was used to determine significance (∗*p* < 0.05; ∗∗*p* < 0.01). Co-IP, Co-Immunoprecipitation; CRY2, cryptochrome 2; HEK293T, human embryonic kidney 293T cell line; PER2, Period 2; WT, wild type.

In the absence of overexpressed PER2, we observed a reduction in the affinity of p.Ser420Phe CRY2 for CLOCK compared to wild-type CRY2 within the BMAL1:CLOCK:CRY2 complex (Fig. 2*A*). PER2 remodels the serine loop of CRY2 and regulates its binding to CLOCK (23, 24). To investigate how PER2 affects the binding between the CRY2 variant and CLOCK, we performed the Co-IP experiment with overexpressed PER2. The results indicated that the CRY2 variant displayed an affinity for CLOCK comparable to that of wild-type CRY2 (Fig. 2*B*). On the other hand, we observed a decreased affinity of the CRY2 variant for PER2 within the complex. Therefore, we proceeded to perform pairwise Co-IP between the CRY2 variant and PER2 or CLOCK. Interestingly, unlike the observed changes in affinity within the complex, we did not detect any significant changes in the binding of the variant to either PER2 or CLOCK (Fig. 2, *C* and *D*).

Co-IP studies indicated that the variant exhibited comparable affinity to the CLOCK:BMAL1 complex in the presence of overexpressed PER2. This, however, does not provide a satisfactory explanation for the observed reduction in repression activity. Given the critical role of the CC–helix in nuclear shuttling, we hypothesized that the lower repressor activity of the variant might result from improper nuclear localization. Consequently, we proceeded to investigate the subcellular distribution of the CRY2 variant.

### Subcellular localization of p.Ser420Phe CRY2

**Figure 3:**
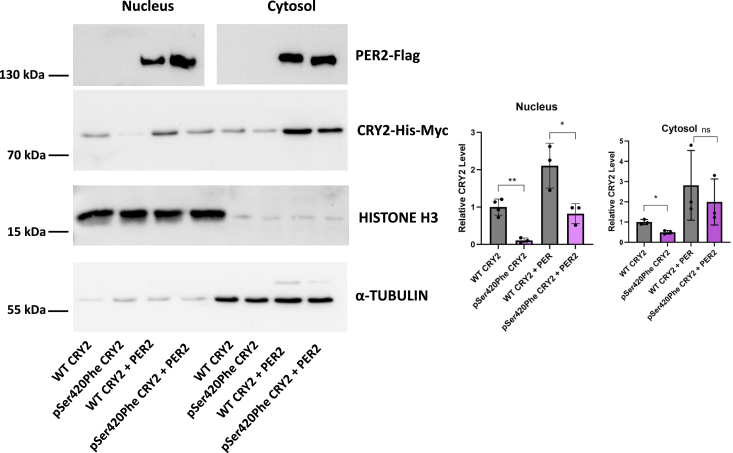
**Subcellular localization of the wild type and p.Ser420Phe CRY2.** Representative Western blot images of nuclear and cytosolic fractions of wild type (WT) and p.Ser420Phe CRY2 in the absence and presence of PER2 overexpression. Transfections of HEK293T cells with 500 ng WT or variant pcDNA-*Cry2*-His-Myc plasmids and 500 ng pFLAG-CMV-*Per2* were performed. The cells were extracted the following day, and subcellular fractionation was conducted. Alpha-tubulin and histone H3 were used as control. The amount of CRY2 was divided by HISTONE H3 for the nucleus and α-TUBULIN for cytosol and normalized based on WT CRY2. Quantification of nuclear (*left*) and cytosolic (*right*) fractions of WT and p.Ser420Phe CRY2 in the absence and presence of overexpressed PER2 (n ≥ 3). Data represent the mean  ±  S.D. of at least three biological replicates. The unpaired *t* test was used for significance analysis (∗*p* < 0.05; ∗∗*p* < 0.01). Separate comparisons were made between WT CRY2 and the variant. CRY2, cryptochrome 2; HEK293T, human embryonic kidney 293T cell line; PER2, Period 2.

We performed subcellular fractionation to separate the nucleus and cytosol. Following cellular fractionation, we initially assessed the purity of the nuclear and cytosolic fractions using antibodies against HISTONE H3 and α-TUBULIN, respectively. The purity of both fractions was confirmed, and subsequently, we quantified the levels of wild-type and variant CRY2 in both the cytosol and the nucleus, in the presence or absence of PER2. The results of this experiment revealed that in the absence of PER2, both cytosolic and nuclear levels of p.Ser420Phe CRY2 were significantly lower than those of wild-type CRY2 (Fig. 3). This suggests that the variant's stability is compromised. Moreover, even in the presence of PER2, the amount of the CRY2 variant in the nucleus remained considerably lower than that of wild-type CRY2. This observation explains why the CRY2 variant was unable to effectively repress CLOCK:BMAL1-driven transcription, even when PER2 was overexpressed.

**Figure 4:**
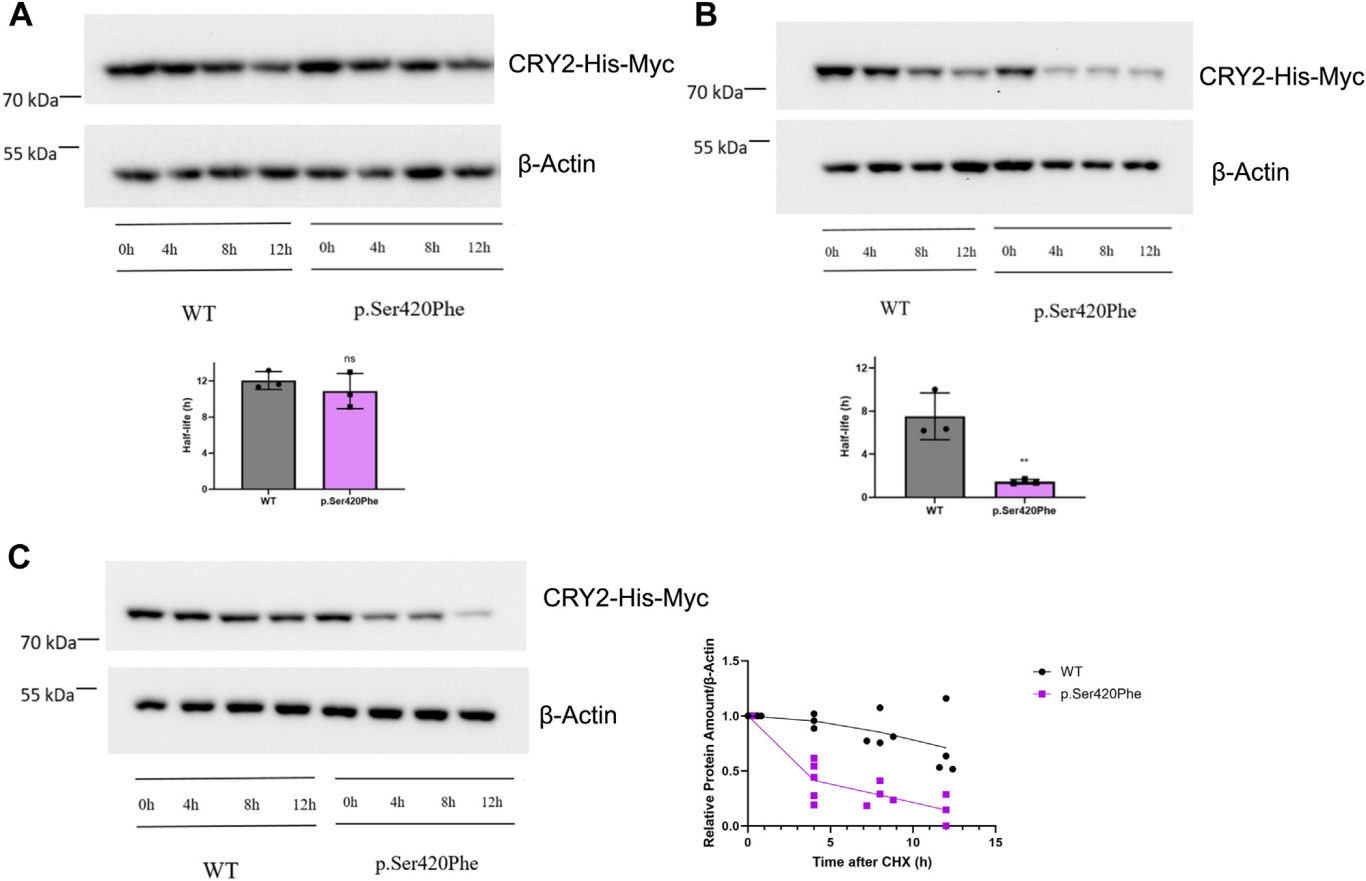
**CHX assay.***A*, representative Western blot images of protein degradation assay of WT and p.Ser420Phe CRY2 in the presence and (*B*) the absence of overexpressed PER2 (n = 3). *C*, representative Western blot image of CHX chase assay of WT and p.Ser420Phe CRY2 with MG132, a proteasome inhibitor treatment. HEK293T cells were transfected with 500 ng WT or variant pcDNA-*Cry2*-His-Myc plasmids, as well as 500 ng pFLAG-CMV-*Per2*. The next day, the cells were treated with 20 μg/ml CHX and 10 μM MG132 to inhibit protein synthesis. The WT and variant CRY2 were detected with anti-Myc, and β-actin were used as a loading control (n = 3). The half-lives of WT and p.Ser420Phe CRY2 were calculated using a one-phase exponential decay function with R^2^ > 0.95. Data represent the mean  ±  SD of three biological replicates. The unpaired *t* test was employed to test for significance (∗∗*p* < 0.01). CHX, cycloheximide; CRY2, cryptochrome 2; h, hour; HEK293T, human embryonic kidney 293T cell line; PER2, Period 2; WT, wild type.

These findings collectively indicate that the CRY2 variant exhibits instability, even when PER2 is present. To further investigate its stability, we carried out a cycloheximide (CHX) chase experiment. HEK293T cells were transfected with either wild-type or variant pcDNA-Cry2-His-Myc plasmids and were then treated with CHX to inhibit protein synthesis, with or without the overexpression of PER2. Cells were harvested at 4-h intervals over a span of 12 h, and their protein extracts were subjected to Western blot analysis. As expected, the presence of overexpressed PER2 increased the stability of wild-type CRY2 (Fig. 4, *A* and *B*). The half-life of CRY2 extended to approximately 12 h when PER2 was overexpressed, whereas it was approximately 7.9 h without PER2 overexpression. Notably, in the presence of overexpressed PER2, the half-life of the CRY2 variant was comparable to that of wild-type CRY2 (Fig. 4*A*). In contrast, without PER2 overexpression, the variant's half-life was approximately 2 h and significantly shorter than wild-type CRY2 (Fig. 4*B*).

**Figure 5:**
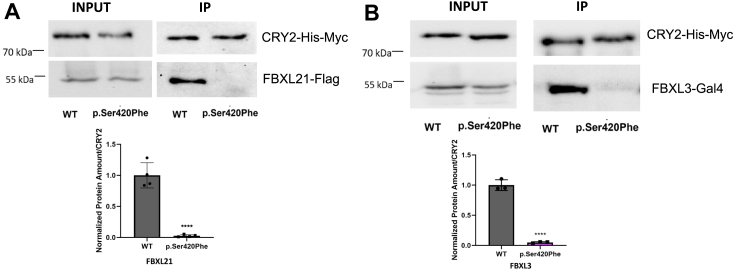
**Determination of the interaction between p.Ser420Phe CRY2 with FBXL21 and FBXL3.***A*, the interaction between WT and p.Ser420Phe CRY2 with FBXL21 and *B*, FBXL3 (n ≥ 3). HEK293T cells were transfected with 500 ng WT or variant pcDNA-*Cry2*-His-Myc plasmids, 500 ng pBind-*FBXL3*-Gal4, and 500 ng pFLAG-CMV-*FBXL21*. The cells were extracted the next day, and Co-IP was conducted with MYC resin. Western blot was used to quantify FBXL21 and FBXL3 and divided by the amount of CRY2, which was normalized based on wild type. To evaluate the significance, the unpaired *t* test was performed (∗∗∗∗*p* < 0.0001). Co-IP, Co-Immunoprecipitation; CRY2, cryptochrome 2; HEK293T, human embryonic kidney 293T cell line; WT, wild type.

Given that CRY proteins are known to undergo ubiquitination by E3 ligases followed by degradation through the proteasome pathway (16, 25), we conducted a CHX chase assay with MG132, an inhibitor of the proteasome complex, to gain insights into the degradation mechanism (Fig. 4*C*). The Western blot results indicated that inhibition of the proteasome led to the stabilization of wild-type CRY2. However, this stabilization effect was not observed in the case of the p.Ser420Phe CRY2 variant, suggesting that this variant is subject to a noncanonical degradation mechanism (Fig. 4*C*). We further explored the possibility that the CRY2 variant may not interact with E3 ligases (FBXL3 and FBXL21), which are essential for proteasome-mediated degradation. To investigate this, HEK293T cells were transfected with plasmids containing either wild-type or variant pcDNA-*Cry2*-His-Myc, along with pBind-*FBXL3*-Gal4 and pFLAG-CMV-*FBXL21*. MYC resin was used to precipitate CRY2-MYC, and the levels of precipitated FBXL3 and FBXL21 in association with CRY2 were determined by Western blot analysis. The results revealed that the interaction between p.Ser420Phe CRY2 and both ubiquitin ligases disappeared (Fig. 5). This observation could provide an explanation for why p.Ser420Phe CRY2 is not subject to proteasome-mediated degradation.

### Lysosomal degradation of p.Ser420Phe CRY2

**Figure 6:**
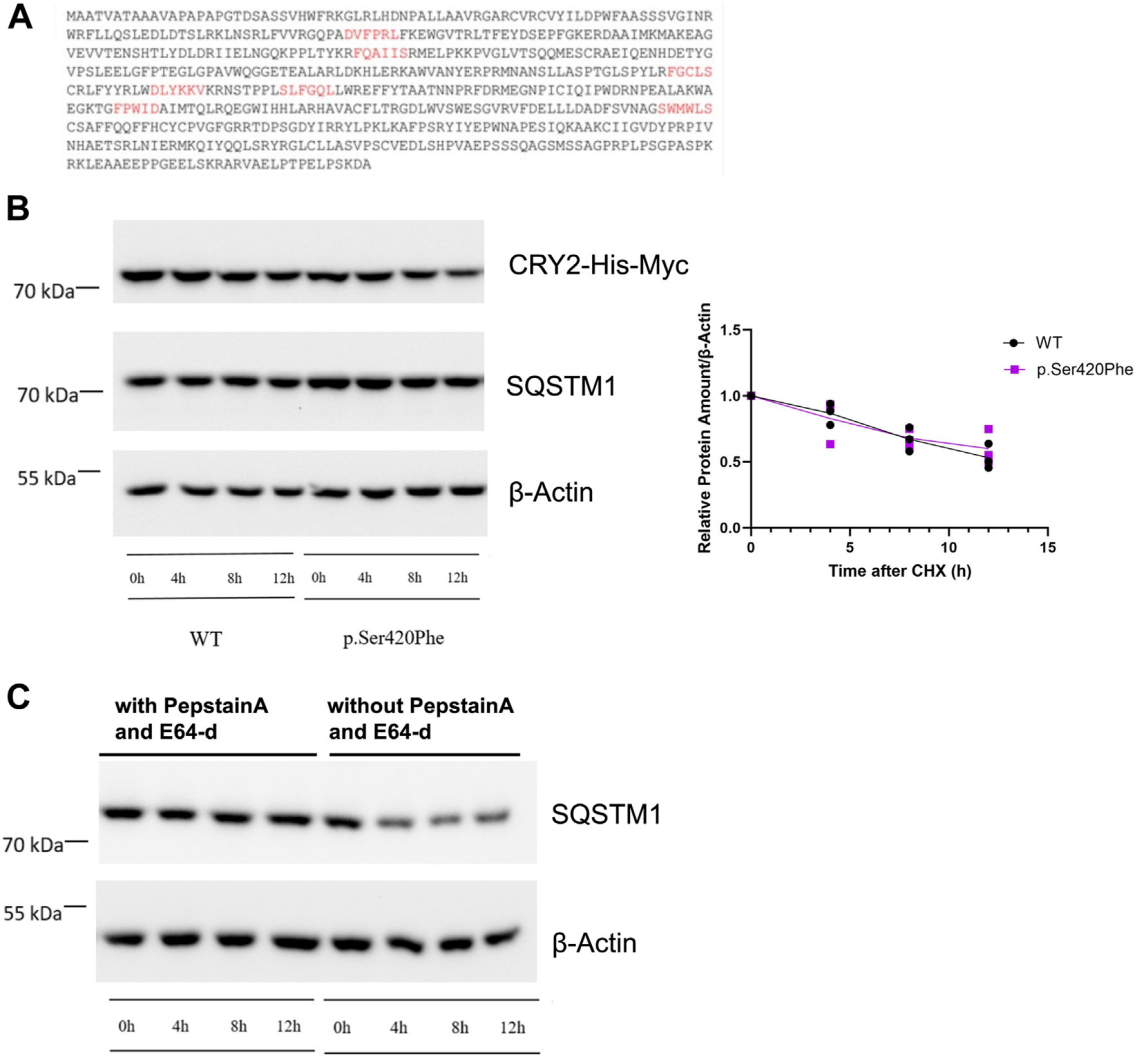
**CRY2 has LIR motifs and the stability of p.Ser420Phe CRY2 is regulated by the lysosome.***A*, presence of putative LIR motifs on human CRY2 protein (UniProtKB/Swiss-Prot: Q49AN0.2). LIR motifs were shown in red. *B*, representative Western blot image of CHX chase assay of WT p.Ser420Phe CRY2 with and (*C*) without lysosome inhibitor treatment. 500 ng WT or variant pcDNA-*Cry2*-His-Myc plasmids were transfected to HEK293T cells. After overnight incubation, the cells were treated with 20 μg/ml CHX to inhibit protein synthesis and 5 μM PepstatinA and 5 μM E64-d to inhibit the lysosome. The WT and variant CRY2 were detected with anti-Myc, β-actin was used as a loading control, and SQSTM1 was used as an indicator of autophagic degradation. *B*, *right panel*, quantification of Western blots (n = 3). The amount of CRY2 was divided by β-actin and normalized based on 0 h. *C*, Western blot image of CHX chase assay of SQSTM1 with and without treating lysosome inhibitors PepstatinA and E64-d. CHX, cycloheximide; CRY2, cryptochrome 2; h, hour; HEK293T, human embryonic kidney 293T cell line; WT, wild type.

A previous study has reported that CRY1 contains multiple LIR (LC3-interacting region) motifs and undergoes degradation through the lysosomal pathway as an alternative to the proteasomal pathway, although this pathway is not observed for CRY2 in mouse liver (20). The LIR motif typically consists of the sequence W/F/Y-X-X-I/L/V, often accompanied by flanking acidic amino acids (E, D, S, T) located at either the N or C terminals (21). In our analysis of the primary human CRY2 sequence, we identified seven putative LIR motifs (Fig. 6*A*; highlighted by red color). To investigate whether both CRY2 and its variant are indeed degraded through the lysosomal pathway, we conducted a cycloheximide (CHX) chase assay in the presence of lysosome inhibitors, PepstatinA and E64-d (26). In contrast to a previous report, degradation of both wild type and CRY2 variant were inhibited with PepstatinA and E64-d (Fig. 6*B*). As a control, we also examined the stability of SQSTM1, which serves as a natural control for lysosomal degradation pathway activity, both in the presence and absence of these inhibitors. The results indicated that SQSTM1 degradation occurred in the absence of inhibitors (Fig. 6*C*).

Collectively, these findings suggest that despite the previously reported absence of the lysosomal degradation pathway in CRY2 in mouse liver, the presence of these LIR motifs indicates that CRY2 in humans is indeed subject to degradation *via* the lysosomal pathway at the cellular level.

### The effect of p.Ser420Phe CRY2 on circadian rhythm at the cellular level

**Figure 7:**
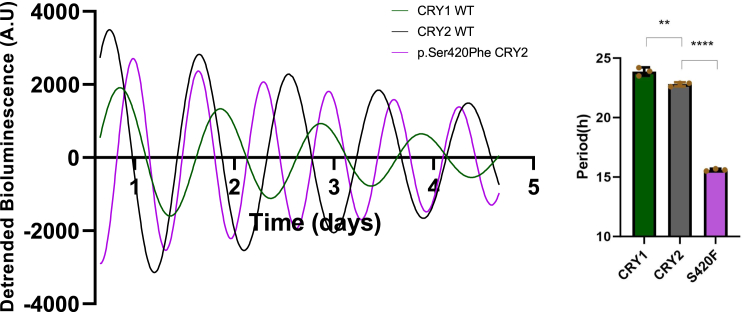
**Real-time bioluminescence rescue assay.** The representative bioluminescence of WT CRY1, WT and p.Ser420Phe CRY2 (n = 3). MEF *Cry1*^−/−^*Cry2*^−/−^ cells were transfected with WT *Cry1*, WT and p.Ser420Phe *Cry2* in pMU2 rescue plasmid and pGL3-*Per2*-d*Luc*. After 72 h, the cells were synchronized with 0.1 μM DXM and the bioluminescence was recorded for 5 days. The raw data were detrended by baseline-subtracting a 24-h running average and, fitted to a sine wave using Lumicycle analysis software (Actimetrics) to determine the periods. Data represent the mean  ±  S.D. of three biological replicates. The unpaired *t* test was employed to test for the significance of the periods (∗∗*p* < 0.01; ∗∗∗∗*p* < 0.0001). CRY, cryptochrome; MEF, mouse embryonic fibroblast; WT, Wild type.

Considering the fact that the p.Ser420Phe CRY2 variant is unable to undergo degradation *via* the canonical pathway but instead undergoes lysosomal degradation, we aimed to comprehend how this differential mechanism might impact circadian rhythm regulation. To investigate this, we assessed the effect of the p.Ser420Phe CRY2 variant on circadian rhythm using *Cry1*^−/−^*Cry2*^−/−^ mouse embryonic fibroblast cells (*Cry* DKO MEF), a cell line established by the Ueda group (27). In our experiments, *Cry* DKO MEF cells were transfected with three different constructs: pMU2-P(*CRY1*)-(intron 336)-*Cry1*, the wild-type pMU2-P(*CRY1*)-(intron 336)-*Cry2*, and the variant pMU2-P(*CRY1*)-(intron 336)-*Cry2*. Additionally, a luciferase reporter, pGL3-*Per2*-dLuc, was introduced to measure bioluminescence levels. Upon expression of both wild-type *Cry1* and *Cry2*, *Cry* DKO MEF cells successfully restored the circadian rhythm, as anticipated. Cells expressing *Cry1* exhibited a longer period length compared to those expressing wild-type *Cry2*, aligning with previous studies (28, 29) (Fig. 7). *Cry* DKO MEF cells expressing the p.Ser420Phe CRY2 variant exhibited a rescued rhythm but with a significantly 7 h shorter period length compared to cells expressing wild-type CRY2 (Fig. 7). These findings strongly suggest that the pathways of lysosomal and proteasomal degradation play crucial roles in determining the period length of the circadian rhythm.

## Discussion

The circadian rhythm, a fundamental biological process that regulates daily physiological and behavioral patterns, is intricately controlled by a molecular clock system. This system relies on interlocking transcriptional/translational feedback loops (TTFLs) established through interactions among core clock proteins (3, 5, 30). Among these core clock proteins, Cryptochromes (CRYs) play a pivotal role in repressing CLOCK:BMAL1-driven transcription, contributing significantly to the maintenance of circadian rhythms. CRYs possess two distinct structural domains: the PHR domain and extended C-terminal domains (5, 19) where the PHR domain is divided into α-helical, α/β, and coiled-coil (CC)-helix subdomains. The intrinsically disordered C-terminal domain modulates the function of the PHR domain (18). The CC–helix interacts directly with the BMAL1 transactivation domain (TAD) and PER2 (13, 31), critically influencing circadian regulation. This interaction is important for the regulation of circadian rhythms and the molecular clock system.

In this study, we have shed light on the importance of the CC-helix of CRY2, not only for its interaction with E3 ligases but also for its degradation through the lysosomal pathway. Our findings reveal intriguing insights into the functional consequences of the p.Ser420Phe CRY2 variant. While this variant exhibits comparable affinity to PER2 as the wild-type CRY2, its affinity with other core clock components in the transcription repressor complex is diminished. This reduced affinity may have a significant impact on its repressor activity, especially considering PER2's role in displacing CRY2-mediated repression (7, 8, 32). Additionally, our study revealed altered subcellular localization in the CRY2 variant, consistent with previous studies where CC–helix deletion led to reduced nuclear translocation (17). The substitution of Ser–420 in the CRY2 variant may influence the conformation of nuclear localization, potentially hindering proper translocation. Consequently, the CRY2 variant may exhibit reduced activity in repressing CLOCK:BMAL1 transactivation, a hypothesis that warrants further validation.

**Figure 8:**
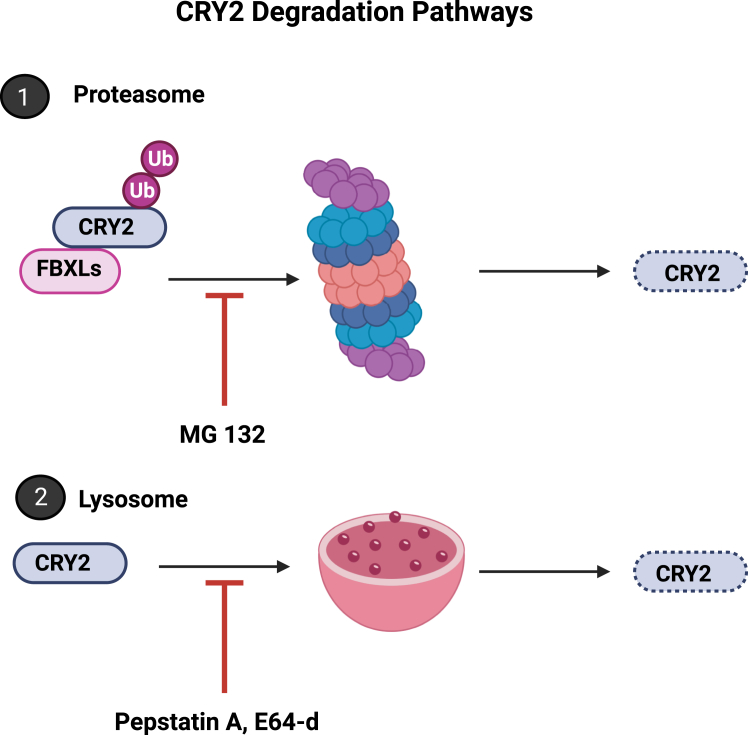
**The depiction of CRY2 degradation pathways.** CRY2 is degraded by two alternative pathways: proteasomal and lysosomal. MG132 inhibits proteasomal degradation. PepstatinA and E64-d inhibit lysosomal degradation.

Our investigations into the degradation mechanisms of the p.Ser420Phe CRY2 variant have uncovered intriguing results. In the absence of PER2, this variant demonstrates instability, prompting speculation about proteasomal degradation. However, it is noteworthy that the variant is unable to bind to E3 ligases FBXL3 and FBXL21, essential for the proteasomal degradation of CRYs (25, 33, 34) (Fig. 5). This suggests that CRY2 may employ an alternative pathway for its degradation. Contrary to previous studies that proposed lysosomal degradation of CRY1 but not CRY2 (20), our CHX chase assays with lysosome inhibitors Pepstatin A and E64–d affirm lysosomal degradation for both CRY2 and its variant (Fig. 4). Several factors may explain the disparity between our observations and the previous study regarding the lysosomal degradation of CRY2: (i) Differences in experimental setups. In our study, we employed HEK 293T cell lines for degradation assays, whereas the prior study utilized mouse liver samples. These distinct experimental setups could lead to variations in degradation pathways. (ii) Compensation of CRYs. The previous study indicated an accumulation of CRY1 upon autophagy inhibition but did not observe a similar effect on CRY2. We propose that the compensatory relationship between CRY1 and CRY2 might have influenced these outcomes. The accumulation of CRY1 could potentially hinder the accumulation of CRY2. (iii) Western blot results are occasionally non-quantifiable due to antibody limitations. Thus, it is conceivable that the antibody utilized in the previous study might not have been capable of detecting changes in CRY2 levels accurately. Ultimately, our findings introduce a novel perspective on CRY2 degradation pathways, as illustrated in Figure 8. These results suggest the complexity of the degradation mechanisms of core clock proteins and highlight the need for further research to fully elucidate these pathways.

Previous studies showed that complementation of *Cry* DKO MEF cells with *Cry1* and *Cry2* genes restored the circadian rhythm with the long and short period length, respectively (28, 29). Notably, the expression of the p.Ser420Phe CRY2 variant in *Cry* DKO MEF cells sustains the circadian rhythm, with 7 h shortened period length compared to cells expressing wild type CRY2. This indicates the critical role of lysosomal degradation in regulating the period length in circadian rhythm.

Our study has broader implications beyond understanding the molecular mechanisms of circadian regulation. Previous research has shown associations between single nucleotide polymorphisms (SNPs) in CRY genes and various diseases (2). For instance, specific CRYs mutations have been linked to sleep disorders, ADHD, and depression (35, 36, 37, 38). Although we functionally characterized the p.Ser420Phe CRY2 variant, no disease correlations were identified in our analysis, possibly due to its rarity. However, these findings highlight the need for *in vivo* investigations to assess the variant's pathogenicity, which could provide crucial insights into its potential implications for circadian-related disorders and contribute to our understanding of the molecular basis of these conditions.

In conclusion, our study has shed light on the functional properties of the p.Ser420Phe CRY2 variant, emphasizing its impact on interactions with core clock proteins, subcellular localization, and degradation pathways. These findings contribute to our understanding of how genetic variations can influence the molecular clock's functioning and provide a foundation for future research in circadian biology and its connections to various health conditions.

## Experimental procedures

### Site-directed mutagenesis

In this study, we employed two distinct mutagenesis techniques to introduce single nucleotide changes in our target plasmids. Firstly, Phusion polymerase-based quick-change mutagenesis was utilized to modify the pcDNA-*Cry2*-Myc-His vector. This process involved the use of specific primers, namely, CRY2-S420F (CTGGATGTGGCTGTTCTGCAGTGC TTTTC) and CRY2-S420F (GAAAAGCACTGCAGAACA GCCACATCCAG), designed to introduce the desired mutation. Each polymerase chain reaction (PCR) reaction mixture consisted of 1X HF Buffer, 200 μM dNTP mix, 0.3 μM forward and reverse primers, 50 ng of the template plasmid, and 0.02 unit/μl of Phusion Hot Start Flex DNA Polymerase (NEB, catalog no.: M0535S), with a final reaction volume of 50 μl. The PCR protocol involved an initial denaturation step at 98 °C for 2 min, followed by 22 cycles of denaturation at 98 °C for 45 s, annealing at 55 °C for 30 s, extension at 68 °C for 10 min, and a final elongation step at 68 °C for 2 min. Subsequently, for the mutagenesis of pMU2-P(*CRY1*)-(intron 336)-*Cry2*, we employed Q5 high-fidelity DNA polymerase-based quick-change mutagenesis. The reaction mixture contained 1X Q5 Buffer Pack, 200 μM dNTP mix, 0.5 μM forward and reverse primers, 50 ng of the template plasmid, 1X Q5 High GC Enhancer, and 0.02 unit/μl of Q5 DNA Polymerase (NEB, catalog no.: M0491S) in a final volume of 25 μl. The PCR protocol for this reaction included an initial denaturation at 98 °C for 2 min, followed by 12 cycles of denaturation at 98 °C for 20 s, extension at 72 °C for 30 s, and a final extension at 72 °C for 4 min. This was followed by 18 additional cycles with altered annealing conditions (98 °C for 20 s, 60 °C for 30 s, and 72 °C for 4 min) and a final elongation at 72 °C for 5 min.

After PCR amplification, the resulting products were purified using a PCR clean-up kit (Macherey Nagel, catalog no.: 740609.50) following the manufacturer's instructions. The purified samples were then subjected to enzymatic digestion with 1 unit of FastDigest DpnI enzyme (Thermo Scientific, catalog no.: FD1703) for 1 h at 37 °C, followed by inactivation at 80 °C for 5 min. Subsequently, 5 μl of the digested PCR product was transformed into TOP10 *E. coli* competent cells, and colonies were cultured. Plasmids were isolated from these colonies using a Nucleospin Plasmid kit (Macherey Nagel, catalog no.: 740588.50) as per the manufacturer's instructions. Finally, the introduced mutations were verified through Sanger sequencing conducted by Macrogen Europe.

### *Per1*-d*Luc* repression assay on CLOCK:BMAL1–driven transactivation

A total of 4 × 10^4^ HEK293T cells (kindly provided by Prof Aziz Sancar from the University of North Carolina Chapel Hill) were reverse transfected in a 96-well opaque plate. The transfection mix consisted of 125 ng of pCMV-Sport6-*CLOCK*, 50 ng of pCMV-Sport6-*Bmal1*, 50 ng of pGL3-m*Per1*:d*Luc* marker, 5 ng of pGL4-*Renilla*, and 20 ng of pFLAG-CMV-*Per2*, along with either the wild type or variant pcDNA-*Cry2*-His-Myc plasmids. To ensure uniform plasmid concentration across all conditions, an empty pcDNA plasmid was also included in the transfection mix. Additionally, to assess CLOCK:BMAL1 transactivation in the absence of the repressor, pcDNA-His-Myc without *Cry* was transfected as a control. This entire transfection process was carried out using polyethyleneimine (PEI) transfection reagent (Polysciences; catalog no.: 23966-1).

For each experimental condition, reverse transfections were performed in at least three replicates to ensure statistical validity. Following transfection, the 96-well plates were incubated for 24 h to allow for gene expression. Subsequently, the expression levels of firefly luciferase and renilla luciferase were quantified using a Fluoroskan Ascent FL microplate reader, employing the Dual-Glo Luciferase Assay System (Promega). The measurements were conducted in accordance with the manufacturer’s recommended procedures to assess the activity of the transcriptional factors and evaluate the impact of the introduced genetic variants.

### Protein extraction from mammalian cells and Western Blot

Methods that are used for protein extraction and Western blot are previously described in (28, 39, 40). Briefly, the cultured cells with 100% confluency were collected with ice-cold phosphate-buffered saline (PBS) (137 mM NaCl, 2.7 mM KCl, 10 mM Na_2_HPO_4_, 1.8 mM KH_2_PO_4_, pH 7.4). Then, they were centrifuged at 4000*g* at 4 °C for 5 min. The pellet was lysed with ice-cold RIPA buffer (150 mM NaCl, 50 mM Tris, 0.1% SDS, 1% Triton X-100, 1% protease inhibitor cocktail (PIC; catalog no.: 78430), incubated 15 min on ice and centrifuged for 20 min at 15,000*g* at 4 °C. The supernatant was collected, and protein concentration was measured using Pierce 660 nm Protein Assay Reagent (Thermo Scientific, catalog no.: XD341295) with an Epoch microplate reader at 660 nm. The lysates were then boiled for 10 min at 95 °C with 4X Laemmli Buffer (250 mM Tris-HCl at pH:6.8, 40% Glycerol, 4% SDS, 0.02% Bromophenol Blue, and 10% freshly added β-mercaptoethanol). The samples were run on SDS-PAGE and transferred to Polyvinylidene fluoride membrane (PVDF, Millipore, catalog no.: IEVH00005) by wet Western Blot transfer. The membrane was blocked with 5% milk solution 0.15% Tris-buffered saline (TBS)–Tween-20 for 1 h and then incubated overnight at 4 °C on the shaker with the proper primary antibodies, washed with 0.15% TBS-T solution 3 times for 5 min, then incubated for 1 h with the suitable secondary antibody at room temperature. The primary antibodies included Anti-FLAG (Sigma Aldrich, catalog no.: SLCJ3741), Anti-Myc (Abcam, catalog no.: ab18185), Anti-β-actin (Cell Signaling, catalog no.: 8H10D10), anti-Histone H3 antibody (Abcam; catalog no.: ab1791), monoclonal anti-α-tubulin antibody (Sigma–Aldrich; catalog no.: T9026), anti-SQSTM1 monoclonal antibody (Abnova, catalog no.: H00008878-M01), anti-Gal4(DBD) antibody (Santa Cruz, catalog no.: sc-577). HRP-conjugated anti-mouse (Santa Cruz; catalog no.: SC-358920), HRP-conjugated anti-rabbit (Cell Signaling; catalog no.: 7074), and rabbit polyclonal IgG antibody (Santa Cruz, catalog no.: sc-48805) were used as secondary antibodies. After the incubation with the secondary antibody, the membrane was again washed with 0.15% TBS-T solution 3 times for 5 min and visualized using fresh homemade ECL (1.25 mM luminol (Sigma, catalog no.: MKCP9100), 0.225 mM p-coumaric acid (Sigma, catalog no.: C9008-25G), 0.1 M Tris (pH = 8.8), H_2_O_2_ (9 × 10^−5^ [v/v]) with BioRad ChemiDoc Touch.

### Protein complex immunoprecipitation (Co-IP)

4 × 10^5^ HEK cells per well were seed to 6-well plate. After 24 h, when the cells reached 70% confluency, they were transfected with 500 ng wild type or variant pcDNA-*Cry2*-His-Myc, 1000 ng Flag-CMV-*Bmal1*, 150 ng Flag-CMV-*CLOCK*, and 350 ng Flag-*PER2*-CMV using PEI. For determining the interaction with FBXLs, HEK293T cells were transfected with 500 ng wild type or variant pcDNA-Cry2-His-Myc plasmids, 500 ng pBind-*FBXL3*-Gal4, and 500 ng pFLAG-CMV-*FBXL21*. After 24 h, the cells were harvested with ice-cold PBS and centrifuged at 4000*g* at 4 °C for 5 min. The pellets were lysed in 300 μl passive lysis buffer (PLB) (15 mM HEPES, 5 mM NaF, 300 mM NaCl, 1% NP-40, and 1% freshly added PIC) and incubated for 15 min on ice. The samples were centrifuged for 20 min at 15,000*g* at 4 °C; then, the supernatants were collected. 10% of the supernatant was saved as input. 10 μl EZview Red Anti-c-Myc Affinity Gel beads per sample (catalog no.: E6654) were equilibrated by washing twice with PLB. Supernatants and equilibrated resins were mixed for 2 h at 4 °C in a vertical rotator. After 2 h, the samples were centrifuged for 30 s at 8200*g* at 4 °C. Supernatants were removed, and beads were washed 3-times with PLB (without PIC). After the final centrifuge, the proteins from the beads were isolated by boiling in Laemmli Buffer, and Protein quantification and detection were carried out using Western blotting, following the procedures outlined in the “Extraction of proteins from cells and Western Blot” section of the experimental protocol.

### Subcellular fractionation

Subcellular fractionations were performed as described previously (41). A total of 4 × 10^5^ HEK293T cells were seeded in a 6-well plate. After 24 h, the cells were transfected with 500 ng wild-type or variant pcDNA-*Cry2*-His-Myc plasmids and 500 ng pFLAG-CMV-*Per2* by PEI. The cells were collected and lysed using cytosolic lysis buffer after 24 h of incubation (10 mM HEPES pH 7.9, 10 mM KCl, 0.1 mM EDTA, 0.05% NP-40, proteasome inhibitor). Then samples were incubated for 15 min on ice and centrifuged for 5 min at 650*g* at 4 °C. The supernatant was saved as the cytosolic fraction, and the pellet was processed for the nuclear fraction. The nuclear fraction was washed twice with cytosolic lysis buffer and centrifuged for 5 min at 650*g* at 4 °C. The pellet was dissolved in nuclear lysis buffer (20 mM HEPES pH 7.9, 0.4 M NaCl, 1 mM EDTA, 10% glycerol, protease inhibitor), then sonicated (60% power, 10 s × 3 cycles) and centrifuged 15,000*g* for 5 min at 4 °C, with the supernatant preserved as the nuclear fraction. The cytosolic fraction was also centrifuged at 15,000*g* for 5 min at 4 °C, the cell debris was removed in the pellet, and the supernatant was preserved as the cytosolic fraction. After determining the protein amount, the fractions were supplemented with Laemmli buffer and protein quantification and detection were carried out using Western blotting, following the procedures outlined in the “Extraction of proteins from cells and Western Blot” section of the experimental protocol.

### CHX chase assay

A 12-well tissue culture plate was prepared with the seeding of 2 × 10^5^ HEK293T cells per well. These cells were allowed to grow until they reached approximately 70% confluency. At this point, transfection was carried out using polyethyleneimine (PEI) as the transfection reagent. Specifically, 500 ng of either wild-type or mutant pcDNA-*Cry2*-His-Myc plasmids and 500 ng of pFLAG-CMV-Per2 plasmids were transfected into the cells. After the transfection, a 24-h incubation period was allowed to ensure gene expression. Subsequently, the cells were subjected to treatment with 20 μg/ml of cycloheximide (CHX) from Sigma (catalog no.: 66819). To monitor the effect of proteasome inhibition, the cells were additionally treated with CHX (20 μg/ml) in combination with 10 μM MG-132 from Sigma (catalog no.: 1211877-36-9). To investigate lysosomal inhibition, a different treatment regimen was applied: the cells were treated with CHX (20 μg/ml) along with 5 μg/ml PepstainA from Sigma (catalog no.: P5318) and 5 μg/ml E64-d (gifted by Prof. Dr Devrim Gözüaçık, Koç University). To analyze the changes in protein levels and their stability over time, cells were collected at 4-h intervals for a total duration of 12 h. The samples obtained immediately after CHX treatment were designated as the 0-h time point. Protein quantification and detection were carried out using Western blotting, following the procedures outlined in the “Extraction of proteins from cells and Western Blot” section of the experimental protocol.

### Real-time bioluminescence rescue assay

4 × 10^5^ MEF *Cry1*^−/−^*Cry2*^−/−^ cells (gifted by Prof Hiroki R. Ueda, RIKEN, Japan) were seeded into 35 mm culture dishes (27). The cells were transfected with 4000 ng pGL3-*Per2*-d*Luc* (luciferase reporter), pMU2-P(*CRY1*)-(intron 336)-*Cry1*, and wild type or variant pMU2-P(*CRY1*)-(intron 336)-*Cry2* using the FuGENE6 transfection reagent (Promega; catalog no.: E2691) after 24 h, when they have reached 70% confluency. After 3 days of incubation, the cells were reset with 0.1 μM dexamethasone (DXM; Sigma Aldrich; catalog no.: D4902). After 2 h of incubation, the medium was replaced with lumicycle medium (10 g Dulbecco’s modified Eagle’s medium powder (Sigma; catalog no.: D-2902), 0.35 g sodium bicarbonate (tissue culture grade; Sigma; catalog no.: S5761), 3.5 g D(+) glucose powder (tissue culture grade; Sigma; catalog no.: G7021), 10 ml 1 M HEPES buffer (Gibco; catalog no.: 15140-122), 2.5 ml penicillin/streptomycin (100 μg/ml), 50 ml 5% fetal bovine serum, and up to 1 L sterile Milli-Q water) with 0.1 μM freshly added luciferin. The plates were then sealed with silicone grease and placed in the LumiCycle 32 Luminometer (Actimetrics). The bioluminescence readings were recorded for 70 s every 10 min for 5 days.

## Data availability

All data generated or analyzed during this study are included in this article.

## Conflict of interest

The authors declare that they have no conflicts of interest with the contents of this article.
